# Posterior-only vs. combined posterior-anterior approaches in treating lumbar and lumbosacral spinal tuberculosis: a retrospective study with minimum 7-year follow-up

**DOI:** 10.1186/s13018-020-01616-7

**Published:** 2020-03-10

**Authors:** Zheng Liu, Penghui Zhang, Weiwei Li, Zhengchao Xu, Xiyang Wang

**Affiliations:** 1 Department of Spine Surgery, Xiangya Hospital, Central South University, 87#Xiangya Road, Changsha, 410008 Hunan People’s Republic of China; 2 Hunan Engineering Laboratory of Advanced Artificial Osteo-materials, Xiangya Hospital, Central South University, 87#Xiangya Road, Changsha, 410008 Hunan People’s Republic of China; 3 The Orthopedics Department, the Seventh Affiliated Hospital, Sun Yat-sen University, 628#Zhenyuan Road, Zhenmei Community, Guangming District, Shenzhen, 518107 Guangdong People’s Republic of China

**Keywords:** Lumbar spinal tuberculosis, Lumbosacral spinal tuberculosis, Posterior-only approach, Combined posterior-anterior approaches, Long-term follow-up

## Abstract

**Background:**

There is no comparative study with long-term follow-up between posterior-only and combined posterior-anterior approaches in treating lumbar spinal tuberculosis (LSTB) and lumbosacral spinal tuberculosis (LSSTB). This retrospective study aimed to compare and evaluate the long-term outcomes of these two surgical approaches in LSTB and LSSTB.

**Methods:**

Thirty patients with LSTB and 12 patients with LSSTB underwent posterior-only approach (group A); 26 patients with LSTB and 14 patients with LSSTB were managed with combined posterior-anterior approaches (group B). Analysis and comparison in clinical and radiographic outcomes between the two groups were performed.

**Results:**

The intra-operative bleeding amount, surgery time, and hospitalization days in group A were less than that in group B (*P* < 0.05). These patients were followed for a minimum of 7 years. All patients achieved completely healing within 2-year follow-up. Bony fusion was obtained in all patients. The visual analog scale, Japanese Orthopedic Association score, Oswestry Disability index, and Kirkaldy-Willis functional evaluation at the final visit demonstrated that all patients in both groups achieved satisfactory results. There was no significant difference in angle correction or maintaining correction between combined posterior-anterior approaches and posterior-only approach (*P* > 0.05). Complications occurred in both groups.

**Conclusions:**

Both combined posterior-anterior approaches and posterior-only approach can achieve satisfactory clinical and radiological outcomes of long-term in treating LSTB and LSSTB, while posterior-only approach can safely and effectively achieve lesion debridement, decompression, and stability reconstruction and maintenance with the advantages of less invasive surgery, less bleeding, shorter surgery time, and hospital stay, and fewer complications. So, posterior-only approach seemed to be superior to the combined posterior-anterior one.

## Background

According to the latest report by the World Health Organization in 2018 [1], the number of new cases of tuberculosis (TB) worldwide exceeded 10 million, and the estimated number of deaths reached 1.6 million in 2017. Among developing countries, China ranked second among the 30 countries with highest TB burden, second only to India.

As a common extrapulmonary TB, spinal tuberculosis (STB) accounted for half of skeletal TB, which often leads to severe damage on health, resulting in a serious social and economic load [2]. In China, lumbar spinal tuberculosis (LSTB) is with most incidence [3], followed by thoracic STB, while lumbosacral spinal tuberculosis (LSSTB) is relatively uncommon. As the area with the maximum spinal load, lumbar and lumbosacral vertebrae are the stress concentration points with high range of motion. Because of the special anatomy in lumbar and lumbosacral junction, STB can lead to vertebral destruction and spinal instability, resulting in varying degrees of kyphosis, or neurological damage, and even paralysis [4].

Anti-TB chemotherapy is still the mainstay of treatment in STB [5]. The cure can be obtained in most patients by strict and standardized anti-TB drug treatment. While for the patients with spinal instability or deformity, severe or progressive neurological dysfunction, and extensive paravertebral or epidural abscesses, surgical management is frequently imperative. The purpose of surgery is not only to debride the lesion, but also to decompress the spinal cord, restore normal spinal alignment, and reconstruct the spinal stability [6, 7]. In recent years, various surgical methods have been described for treating LSTB and LSSTB. However, the surgical approach in treating LSTB and LSSTB is still controversial. The anterior approach has been preferred traditionally, which enables direct exposure of the infected lesion [8, 9]. Combined anterior-posterior approaches can overcome the stability-related drawbacks that the anterior approach used alone [10, 11]. Single-stage posterior approach has been described more frequently because of the single incision and less trauma [4].

Although the above surgical methods have their respective advantages and disadvantages, no comparative study in these surgical approaches for LSTB and LSSTB has been performed with long-term follow-up outcomes.

Therefore, the present study was performed to compare the clinical and radiological outcomes in long-term follow-up of posterior-only approach and combined posterior-anterior approaches in treating LSTB and LSSTB.

## Materials and methods

### Basic information

From January 2002 to September 2011, patients with LSTB or LSSTB who admitted in our hospital were initially reviewed retrospectively. The surgery was performed when any of the following indications was met [4]: (1) significant or progressive radiculopathy or syndrome of cauda equina due to compression from tuberculosis lesion, (2) severe bone destruction with spinal instability or pathological dislocation or developing spinal deformity, (3) vertebral TB lesion with formation of large sequestrum or cavity, and (4) persisting back pain resulting from STB lesion after expectant treatment.

The inclusion criteria should also include all the following conditions: (1) the lesion involved one or two adjacent lumbar unit segment (L1-S1); (2) underwent surgical treatment with posterior-only approach or combined posterior-anterior approaches; (3) definitive diagnosis of TB by pathological examination; (4) with a minimum 7-year follow-up.

Exclusion criteria were as the followings: (1) multi-level large paravertebral abscesses or huge ilio-psoas abscesses; (2) a history of lumbar surgery and/or other spinal diseases affecting the postoperative evaluation, such as adolescent scoliosis or ankylosing spondylitis; (3) severe vertebral osteoporosis on radiographs; (4) loss of follow-up data for any reason, including loss to follow-up and death.

Comprehensive assessment of clinical symptoms, laboratory results, and imaging findings were necessary to diagnose STB. The main clinical symptoms included low back pain, radiating pain, dysesthesia, and dyskinesia of lower limb. Systemic toxicity symptoms, such as fever, night sweats, anorexia, and weight loss, were also presented in some patients. Imaging examinations were routinely performed. The lumbar or lumbosacral imaging findings, such as collapsed vertebrae and necrotic discs, kyphotic deformity, cold abscess formation, and dura compression, were displayed on preoperative radiographs, computed tomography (CT) scans, or magnetic resonance images (MRI). Local lumbar or lumbosacral deformity angle was measured from radiography. The deformity angle was measured by drawing two lines on a lateral image: one on the superior surface of the uppermost-involved vertebra and the other through the inferior surface of lower most-involved vertebra [12]. Pain severity was evaluated using a visual analog scale (VAS). The Japanese Orthopedic Association (JOA) score [13] and Oswestry Disability index (ODI) [14] were applied to evaluate dysfunction and quality of life. University of California at Los Angeles (UCLA) grading scale (Table 1) [15] was used to evaluate the adjacent segment degeneration (ASD) on radiograph.

**Table 1:** Arthritis grade for intervertebral disc degeneration (UCLA classification)

Grade	Disc-space narrowing	Osteophytes	End plate sclerosis
I	−	−	−
II	+	−	−
III	±	+	−
IV	±	±	+

The grade is based on the most severe radiographic finding evident on plain radiographs. Patients were rated based on the worst category satisfied.

*+* present, *−* absent, *±* either present or absent

### Preoperative management

Preoperatively, all patients received standard anti-TB chemotherapy with HRZE for at least 2 weeks, including rifampicin (450 mg/day), isoniazid (300 mg/day), ethambutol (750 mg/day), and pyrazinamide (750 mg/day). If patients accompanied with anemia and hypoproteinemia, nutrition support was necessary and should be strengthened. It was time to carry out the surgery when erythrocyte sedimentation rate (ESR) and C-reactive protein (CRP) had significantly decreased, and constitutional symptoms had obviously relieved.

### Surgical procedures

**Fig.1:**
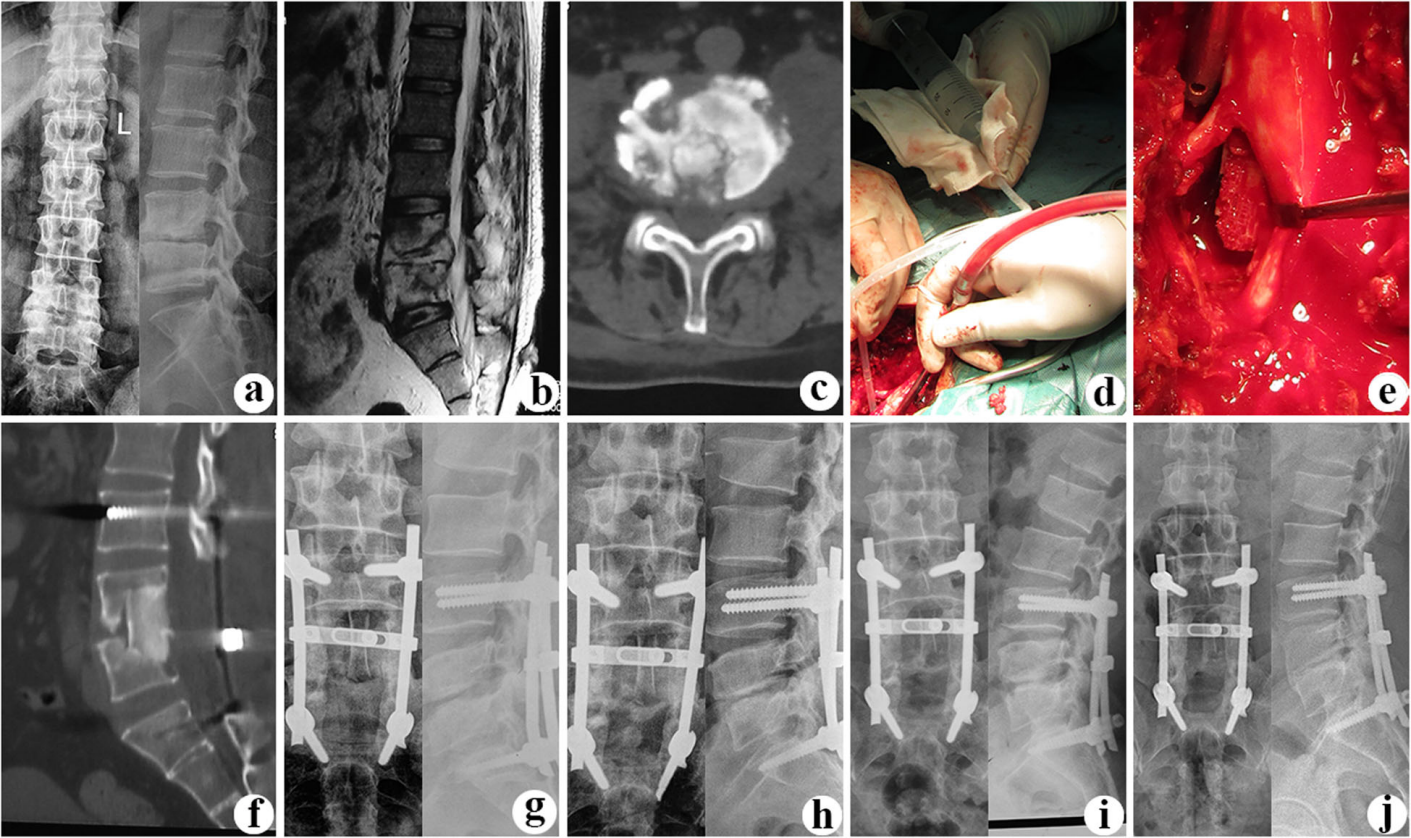
A 36-year-old male with L4/5 lesions underwent the surgical treatment of posterior-only approach. Preoperative imaging data (**a** radiography antero-posterior and lateral, **b** T-2 MRI lateral, and **c** CT cross section) showed L4/5 vertebral bodies’ destruction with paravertebral abscess formation and dural sac compression. **d** Intraoperative photo of pressurized washing and negative-pressure suction (using a suitable flush tube with saline plunged into the depths of the lesion). **e** A trimmed allogeneic block bone was inserted in the interbody to reconstruct the vertebral body. **f**, **g** Postoperative CT and X-ray showed implanted bone and fixation were in good position. **h** At the 8-month follow-up, postoperative radiography showed trabecular bone formation. **i** Satisfied sagittal sequence and good bone fusion in lumbar were achieved at 48-month follow-up. **j** At the final follow-up (91 months after surgery), radiograph illustrated solid bony fusion and no obvious correction angle loss, without signs of fixation failure

**Fig. 2:**
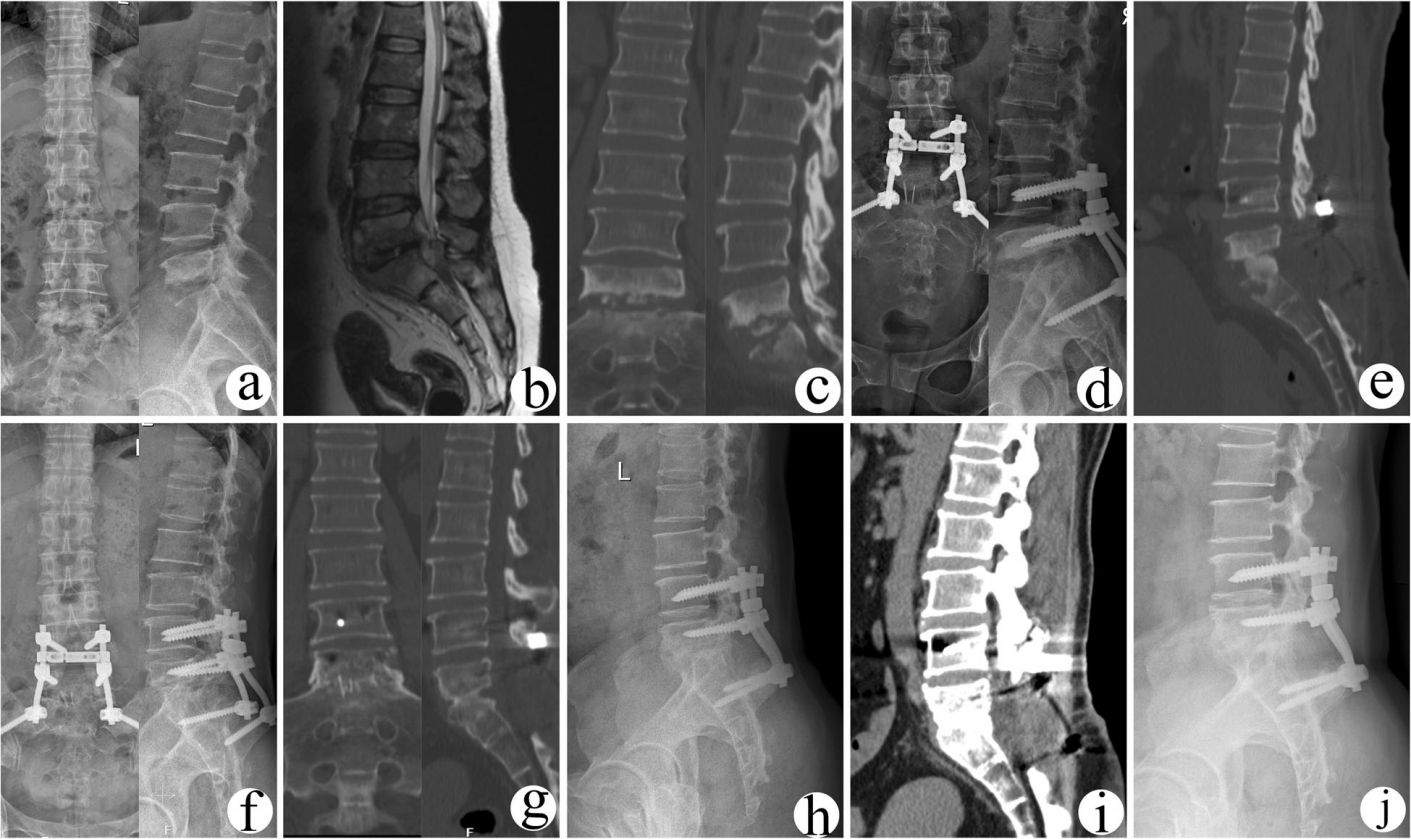
A 47-year-old female with L5/S1 lesions was managed by surgery with posterior-only approach. The preoperative imaging data (**a** X-ray, **b** CT, and **c** MRI) showed severe vertebral destruction and dural sac compression at L5/S1. The postoperative (**d**) radiography and (**e**) CT showed that internal fixation instrument and grafted bone were in good position. **f** The X-ray indicated solid bony fusion at 12 months after surgery. At the 36 months after surgery, (**g**) CT and (**h**) radiography showed solid bony fusion without signs of fixation failure. At the final follow-up (86 months after surgery), (**i**) CT and (**j**) radiograph illustrated solid bony fusion and no obvious angle loss with good fixation position

Patients in group A (posterior-only approach) (Figs. 1 and 2) were placed in prone position. The key surgical procedures were as following. Pedicle screws were implanted in the adjacent one or two normal vertebrae adjacent upper and lower to the pathologic segment. Pedicle screws were inserted in the ilium in cases where the S1 vertebra was severely destructed. Whether fixed to the pathologic vertebrae were based on the extent of vertebral destruction and pedicle integrity. After installing a temporary internal fixation instrument on opposite side to avoid nerve injury during contralateral focal debridement, an expanded hemi-laminectomy or complete laminectomy was performed on the more severely affected side. The superior and inferior articular processes of the vertebrae were partially removed in the same side to expose the intervertebral space. The removal of lesions, including TB granulation tissue, caseous necrotic material, sequestra, abscesses, and necrotic discs and endplates, was performed using curettes of different sizes and angles until scraping of the bone surface produced bleeding. By pressurized washing and negative-pressure suction (using a suitable flush tube with saline plunged into the depths of the lesion) (Fig. 1), all potential residual lesions were debrided as radically as possible. Before tightening the rods, deformities were corrected by compressing and stretching the internal fixation instrument. A trimmed allograft or autograft bone block was inserted in the interbody to reconstruct the vertebral body (Fig. 1). The space between the decorticated transverse processes was carpeted with autogenous or allogeneic particulate bone to promote bone fusion. Local anti-TB therapy with streptomycin (1 g) and isoniazid (0.3 g) was routinely administered in the lesion area. Drainage was routinely established before suturing the incision closed. Each patient’s debrided specimen underwent mycobacterial culture and histopathological examination.

**Fig.3:**
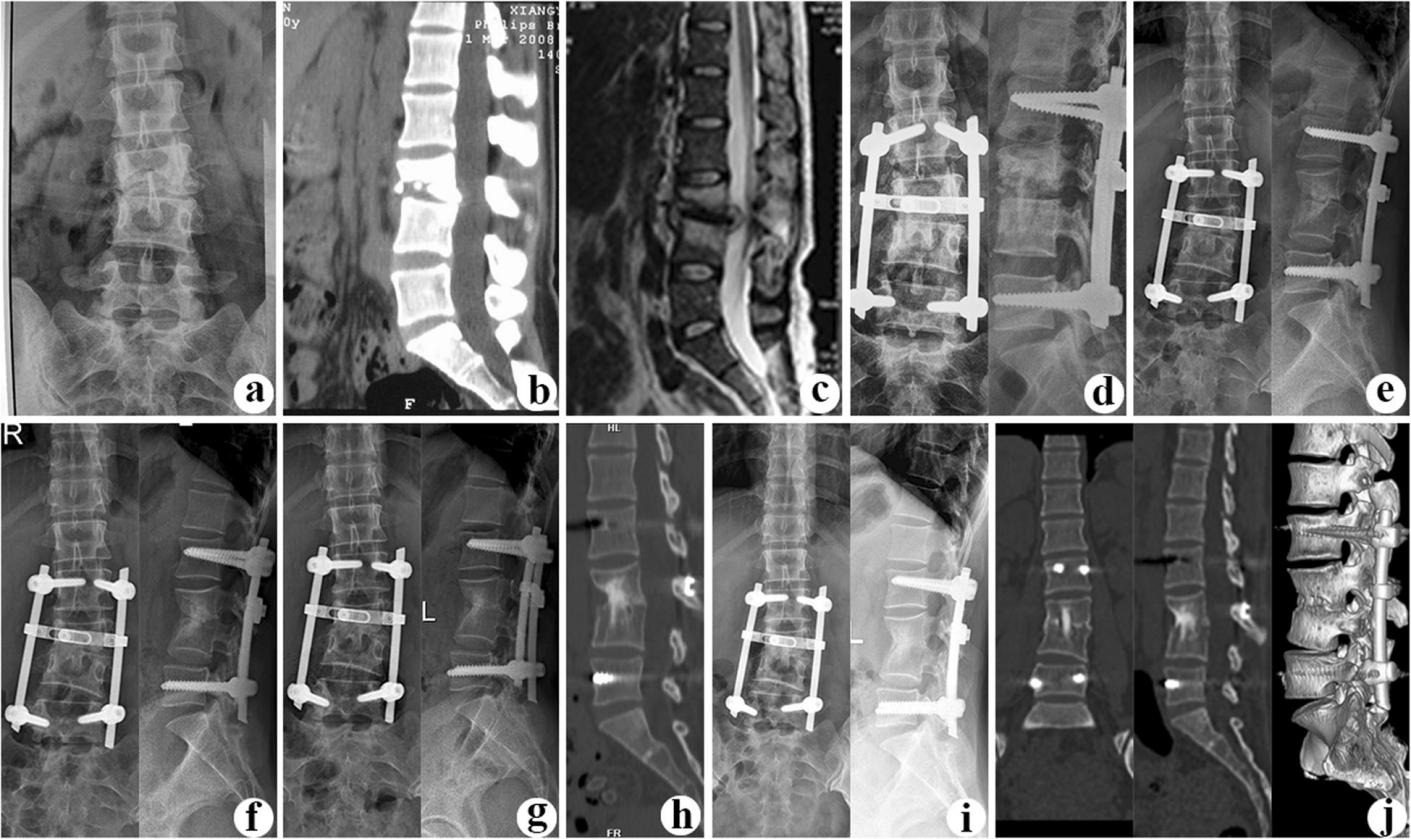
A 27-year-old female with L3/4 lesions underwent surgery by combined posterior-anterior approaches. The preoperative images (**a** X-ray antero-posterior, **b** CT lateral, and **c** T-2 MRI lateral) showed L3/4 vertebral bodies’ destruction with spinal cord compression and kyphosis (16.7°) deformity. Postoperative X-ray (**d**) showed that internal fixation instrument was in good position, and kyphosis had been relieved significantly (7.7°). During the follow-up, at **e** 36 months, **f** 60 months, and **g**, **h** 84 months after surgery, X-ray or CT presented solid bone fusion without signs of fixation failure. **i**, **j** At the last visit (122 months after surgery), radiograph and CT illustrated strong bony fusion and no obvious correction angle loss (1.9°) with good fixation position

**Fig. 4:**
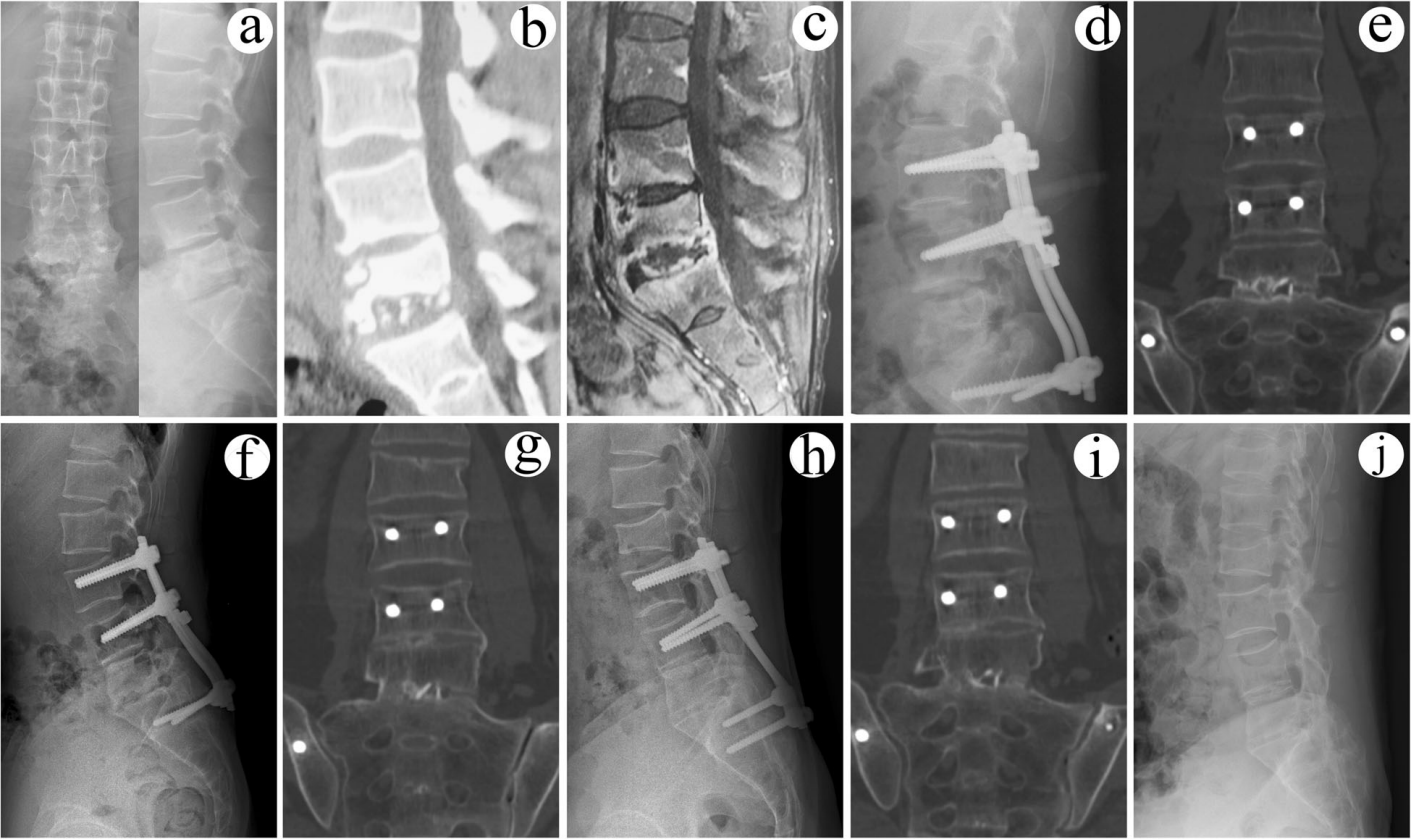
A 28-year-old male with L5/S1 lesions was managed by surgery with combined posterior-anterior approaches. The preoperative imaging data (**a** X-ray, **b** CT, and **c** MRI) showed severe vertebral destruction at L5/S1. The postoperative (**d**) radiography showed that internal fixation instrument was in good position. **e** CT and **f** X-ray indicated solid bony fusion at 12 months after surgery. At the 48 months after surgery, **g** CT and **h** radiography showed solid bony fusion without signs of fixation failure. At the final follow-up (96 months after surgery), **i** CT illustrated solid bony fusion and no obvious angle loss with good fixation position. **j** The radiograph showed the removal of internal fixation instrument and satisfied sagittal sequence

In group B (combined posterior-anterior approaches) (Figs. 3 and 4), posterior fixation as group A without bone grafting was performed first. Then, anterior debridement and fusion was followed. In anterior procedure, patients were placed in the lateral decubitus position. The extra peritoneal anterolateral approach was used, avoiding peritoneal injury. The TB lesions, including paravertebral and ilio-psoas abscess, collapsed the intervertebral disc, and other necrotic tissues were completely debrided until to the surface of healthy and bleeding bone and tissue. Following sufficient debridement, a trimmed allograft or autograft bone block was in the interbody to reconstruct the vertebral anterior column and restore normal height. The other procedures were the same as that in group A.

The same team of doctors reviewed the surgical indications and performed the operations.

### Postoperative management

The postoperative drainage tube was retained until the drainage flow was < 30 ml/day. Intravenous anti-infection and nutritional agents were routinely administered. A standard anti-TB regimen with HRZE was administered for 3 months. The pyrazinamide was then discontinued, and the HRE chemotherapy was continued for 9 to 15 months. Because of the potential adverse effects of anti-TB drugs, hepatic and renal function was regularly evaluated. Two weeks after surgery, gradual walking was allowed with the aid of a spinal orthosis. During the next 3 to 6 months, the orthosis was removed when interbody bony callus formation was evident on imaging examinations.

### Follow-up evaluation

Outpatient visits or telephone follow-up was used for evaluation during the follow-up. The ESR and CRP concentration were measured to assess the activity of the TB lesion during the perioperative period and at 3 months postoperatively. During the follow-up, the position of the graft and instrumentation were investigated by a routine radiography or CT, which was also performed to evaluate the graft fusion status during follow-up according to the modified radiological criteria established by Lee et al. [16]. The local deformity angle, JOA score, ODI, and VAS score were recorded preoperatively, postoperatively, and at the final follow-up. At the final visit, Kirkaldy-Willis functional outcomes [17] were used to evaluate the patients’ living and working conditions. UCLA grading scale was applied to assess ASD on radiograph.

### Statistical analysis

The statistical analysis in this study was performed by the SPSS 24.0 statistical software (IBM Corp., Armonk, NY, USA). Student’s *t* test was used to compare the clinical data between the two groups. A paired *t* test was applied to compare the changes in indices in each group preoperatively, postoperatively, and during follow-up. Any discrepancy in the normal distribution was analyzed using the rank sum test. A *P* value of < 0.05 was considered statistically significant.

## Results

### Patients information

According to the screening criteria, a total of 136 non-consecutive patients were retrieved. Complete follow-up data were available for 82 patients (60%), and 54 patients (40%) were lost to follow-up.

Therefore, a total of 82 patients were enrolled in this study and were divided into two groups (group A and B) according to different surgical approaches. There were 42 patients (24 males and 18 females) with a mean age of 40.3 years (range 19 to 56 years) in group A, and patients were performed with single posterior debridement, bone graft fusion, and fixation (posterior-only approach). In group B, there were 40 patients (21 males and 19 females) with a mean age of 42.5 years (range 18 to 55 years). All of them were managed with one-stage anterior debridement, bone grafting fusion, and posterior instrumentation (combined posterior-anterior approaches).

According to the involved anatomical segment, group A and B were divided into two subgroups respectively: group A1 (30 cases) and B1 (26 cases) were LSTB groups (L1~L5); group A2 (12 cases) and B2 (14 cases) were LSSTB groups (L5~S1).

No active lung TB or HIV positive was found in any patient. The active period of TB was confirmed by an increased ESR and CRP concentration in all patients preoperatively.

### Outcomes

**Fig. 5:**
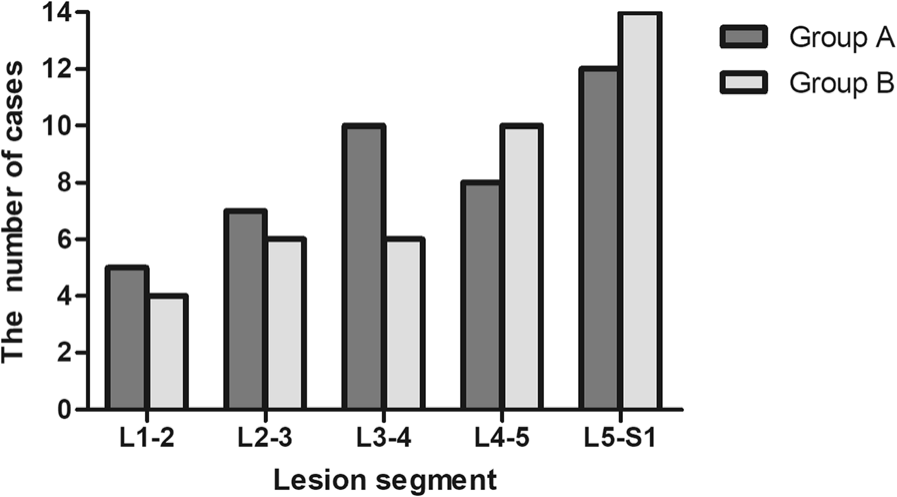
Lesions involved the spinal segment

The clinical data of patients in two groups are shown in Table 2 and Fig. 5. The intra-operative bleeding amount, surgery time, and hospitalization days were lower in group A than B (*P* = 0.000). All patients achieved complete cure within 2-year follow-up [3].

**Table 2:** The clinical data of patients in two groups

	Group A (*N* = 42)	Group B (*N* = 40)	*P* value
Blood loss(ml)	660.7 ± 116.4	963.3 ± 93.1	0.000 < 0.05
Operation time(min)	166.2 ± 18.9	237.0 ± 22.4	0.000 < 0.05
Hospitalization (days)	14.1 ± 2.7	18.6 ± 2.6	0.000 < 0.05
Duration of follow-up (months)	97.9 ± 11.8	100.1 ± 13.3	0.294
Fusion time (months)	8.5 ± 1.6	8.2 ± 1.4	0.364
ESR (mm/h)			
Pre	65.1 ± 7.7	65.8 ± 8.7	0.718
TMP	10.1 ± 2.9	9.5 ± 2.7	0.322
CRP (mg/l)			
Pre	41.5 ± 12.9	44.2 ± 9.9	0.311
TMP	5.0 ± 1.7	5.3 ± 1.8	0.441

*Pre*, preoperative, *TMP* 3 months postoperative, *FFU* final follow-up

The mean follow-up was 99 months (range 84~123 months) in a total of 82 patients. All patients achieved the definitive bony fusion criteria [16] according to radiographic and/or CT assessment during the follow-up. The difference in the mean fusion time was not significant between the two groups (*P* > 0.05). In both groups, normal level was achieved in ESR and CRP within 3 months postoperatively.

The evaluation of pain and dysfunction are shown in Table 3. The VAS, JOA score, and ODI achieved improvement at the 3 months after surgery and the final follow-up in both groups (*P* = 0.000), and there was no significant difference between the two groups (*P* > 0.05). Kirkaldy-Willis functional evaluation at the final visit demonstrated that all patients in both groups achieved excellent or good results.

**Table 3:** The evaluation of pain and dysfunction

	Group A (*N* = 42)	Group B (*N* = 40)	*P* value
VAS			
Pre	7.1 ± 0.8	7.1 ± 0.7	0.978
TMP	2.5 ± 0.5	2.6 ± 0.5	0.796
FFU	0.9 ± 0.4	0.9 ± 0.6	0.798
JOA			
Pre	12.8 ± 3.4	13.2 ± 4.1	0.864
TMP	17.5 ± 4.7	17.9 ± 4.3	0.642
FFU	25.1 ± 2.6	25.6 ± 3.2	0.686
ODI			
Pre	39.2 ± 6.3	38.6 ± 8.8	0.732
TMP	18.3 ± 4.6	17.8 ± 6.4	0.813
FFU	7.2 ± 1.2	7.4 ± 2.4	0.694
Kirkaldy-Willis criteria			
Excellent	32	29	
Good	10	11	
Fair	0	0	
Poor	0	0	

*Pre* preoperative, *TMP* 3 months postoperative, *FFU* final follow-up

Comparison of the local lumbar or lumbosacral deformity angle in group A (A1 and A2) and group B (B1 and B2) is showed in Table 4. According to radiographic or CT measurement, 36 patients (A1_*k*_ and B1_*k*_) had a local lumbar kyphosis angle while 20 patients (A1_*l*_ and B1_*l*_) had a local lumbar lordotic angle. Group A and group B showed no significant differences in the local lumbar or lumbosacral deformity angle preoperatively, immediately postoperatively, or at the last follow-up (*P* > 0.05). In both groups, the local lumbar or lumbosacral deformity angle was significantly improved compared with preoperative angle (*P* = 0.000). The difference in the angle correction rate between groups A and B was not significant (*P* > 0.05).At the final follow-up, the correction loss was (1.3 ± 0.6)° and (1.2 ± 0.6)° in group A1_*k*_ and B1_*k*_ (*P* > 0.05) , (0.9 ± 0.4)°and (0.9 ± 0.3)° in group A1_*l*_ and B1_*l*_ (*P* > 0.05), and (0.7 ± 0.3)°and (0.8 ± 0.3)° in group A2 and B2 (*P* > 0.05), respectively.

**Table 4:** Comparison of the local lumbar and lumbosacral deformity angle

		Kyphosis angle(°)			Angle correction		Correction loss (°)
Group	*n*						
		Pre	Post	FFU	Post (°)	Rate (%)	
A1_*k*_	20	16.8 ± 8.5	3.8±1.5	5.1 ± 1.6	13.1 ± 7.6	74.9 ± 11.8	1.3 ± 0.6
B1_*k*_	16	17.5 ± 8.3	4.3±1.8	5.5 ± 1.8	13.2 ± 7.2	72.0 ± 11.9	1.2 ± 0.6
*P* value		*P* = 0.806	*P* = 0.355	*P* = 0.460	*P* = 0.957	*P* = 0.469	*P* = 0.597
		Lordotic angle (°)					
		Pre	Post	FFU			
A1_*l*_	10	9.7 ± 8.8	16.9 ± 6.0	15.9 ± 6.0	7.1 ± 4.1	47.8 ± 27.2	0.9 ± 0.4
B1_*l*_	10	10.5 ± 7.6	18.1 ± 3.4	17.3 ± 3.7	7.6±5.1	46.4 ± 32.8	0.9 ± 0.3
*P* value		*P* = 0.831	*P* = 0.565	*P* = 0.554	*P* = 0.818	*P* = 0.915	*P* = 0.847
		Lumbosacral angle (°)					
		Pre	Post	FFU			
A2	12	13.4 ± 6.8	19.4 ± 4.3	18.7 ± 4.4	6.0 ± 3.4	34.3 ± 21.5	0.7 ± 0.3
B2	14	13.7 ± 6.2	20.0 ± 3.4	19.3 ± 3.2	6.3 ± 3.2	34.1 ± 20.9	0.8 ± 0.3
*P* value		*P* = 0.884	*P*= 0.676	*P* = 0.703	*P* = 0.832	*P* = 0.985	*P* = 0.611

*Pre* preoperative, *post* postoperative immediately, *FFU* final follow-up

A1_*k*_ and B1_*k*_: the cases with a local lumbar kyphosis angle in lesions

A1_*l*_ and B1_*l*_: the cases with a local lumbar lordotic angle in lesions

### Complications

#### Short-term

In group A, anti-TB treatment intake and TB that presented as a psoas abscess formation in one patient, healed by regular chemotherapy combined with CT-guided percutaneous abscesses drainage and focal catheter infusion. Cerebrospinal fluid leakage occurred in two patients and was treated by leaving the drainage tube a longer time.

In group B, three patients suffered incision superficial infection and were treated by anti-infection and improved with incision dressing change and care. Fistula formation occurred in one patient at 1 week after surgery, which was healed by local anti-TB treatment. Liver function damage induced by anti-TB drugs appeared in one patient, who was treated by a modified chemotherapy combined with hepatic protection drugs (reduced glutathione and magnesium isoglycyrrhizinate). One patient who suffered from angiorrhexis in anterior approach was treated by vascular ligation intraoperatively. Postoperative ileus happened in one patient and was healed with purge treatment.

#### Long-term

According to the UCLA grading scale, all patients were in grade I preoperatively. At the last visit, in group A, 39 patients were in grade I, one patient was in grade II, and two patients were in grade III that showed osteophytes. In group B, 38 patients were in grade I, one patient was in grade II, and one patient was in grade III that manifested as disc-space narrowing and osteophytes. There was no significant difference between the two groups (*P* > 0.05).

## Discussion

The lumbar column is the region with the greatest load and mobility in spine. The lumbosacral segment is a transitional area from mobile lumbar lordosis to fixed sacral kyphosis. The pressure and shear force on lumbar and lumbosacral spine are large, which is easy to cause lumbar instability, abscission of bone graft, and non-fusion postoperatively, resulting in the formation of false joints [18]. The local adjacent anatomical structures of lumbosacral column are complex, including major blood vessels, nerves, and ureters.

LSTB and LSSTB would destroy the anterior and middle columns of the lumbar vertebra and change the physiological lordosis and biomechanics. Anti-TB chemotherapy is not effective to restore and correct the destructed vertebral body. Therefore, it has become a trend to perform surgical treatment associated with anti-TB chemotherapy in the treatment of LSTB and LSSTB [19]. Surgical treatment for LSTB and LSSTB is effective to shorten the treatment period, remove the lesion, restore the height of the affected vertebral body, reconstruct the stability of lumbar and lumbosacral junction, reduce the disability rate, and improve the quality of life [11, 20].

Anterior-only approach has been advocated by many surgeons due to its advantages in adequate lesion debridement under a direct vision [21–25]. However, the shortcomings of anterior approach cannot be ignored. First, kyphosis correction in this approach is insufficient, and great loss of correction postoperatively may occur [26]. Second, especially in lower lumbar and lumbosacral segments, anterior internal fixation is difficult to perform due to the special anatomic characteristics and positions of these segments [27]. Third, because of the complicated anatomy anterior to the lumbar and lumbosacral vertebrae, including major blood vessels, nerves, and ureters, it brings high risk of injury to previous anatomical structure for surgeons to carry out the anterior approach [28]. In addition, complications including postoperative ileus, retrograde ejaculation in males, and ureteral injury are often related with an anterior approach [29].

With the development of posterior pedicle screw and rod instrumentation, combined anterior-posterior approaches and posterior-only approach became new methods to treat STB. Posterior instrumentation, providing three-column and long-segmental fixation, which is stronger than two-column instrumentation in anterior approach, has an obvious superiority in spinal stability reconstruction and kyphotic deformity correction. Ma et al. [23] suggested that posterior fixation has an advantage over the anterior fixation to correct deformity and maintain that correction for the treatment of thoracic and lumbar tuberculosis, particularly for multi-segment STB. Wang et al. [30] reported that in treating thoracic STB, either posterior-only or combined posterior-anterior approaches were superior to anterior approach in correction rate and loss of lordosis angle.

In this study, minimum 7-year long-term follow-up outcomes were achieved to compare the posterior-only approach and combined posterior-anterior approaches in treating STB. Some scholars proposed the surgical procedure of one-stage anterior debridement, bone grafting fusion, and posterior instrumentation in treating STB. The approaches combined the advantages of radical debridement in anterior approach and strong fixation in posterior approach [10, 31]. But the disadvantages of longer operation time, more intraoperative bleeding, and more surgical trauma cannot be ignored.

Posterior-only approach in treating STB has been reported by many scholars [28, 32, 33]. The controversy of whether the damage of posterior column would increase spinal instability is concerned by many surgeons. In our opinion, however, the strong fixation from three-column fixation can effectively maintain short-term spinal stability after surgery. Furthermore, strong bony fusion can be obtained by the combination of interbody bone grafting and lateral bone grafting or posterior lamina reconstruction, keeping long-term spinal stability. In this present study, there was no difference between group A and group B in deformity correction and loss of lordosis angle at the final follow-up. Besides, other advantages of posterior-only approach are as following. First, surgical procedures in posterior-only approach including lesion debridement, bone grafting, and fixation, can be accomplished at one incision without changing the position, which is less invasive. In the present study, the surgery time, bleeding volume, and hospital days were lower in group A than in B. Second, posterior-only approach is familiar to spinal surgeons, avoiding possible injury of the large blood vessels, nerves, or other anatomical structures by the anterior approach.

The controversy that whether posterior approach can thoroughly remove the lesion resulted from that the TB lesions mainly concentrated in the anterior column. Indeed, posterior-only approach offers no advantage on debridement. However, in our experience, removal of lamina and facet joints, then moderate stretch of nerve roots and dura mater can provide adequate surgical space, in which 360° lesion debridement under direct vision can be achieved. In addition, the following procedures, including saline irrigation at lesion site with pressurized washing and negative pressure suction, and postural drainage postoperatively, can effectively drain pus and eliminate residual lesions [34]. Moreover, the cleared lesion can facilitate the penetration of anti-TB drugs, which would improve the efficacy of local anti-TB drugs intraoperatively and systemic anti-TB drugs postoperatively, resulting in healing through spontaneous fusion in STB lesions. Therefore, lesion debridement in posterior-only approach can be sufficient [11].

In the previous studies using posterior-only approach for LSTB or LSSTB, Zeng et al .[20] reported that the correction of lumbosacral angle was 7.9 ± 1.5°, with an average loss of 1.2 ± 0.82° at the last follow-up. Xu et al. [11]reported that the correction loss of lumbar kyphosis angle was 15.4 ± 5.0°, with a mean loss of 1.2 ± 0.82°.

These results from the previous studies are similar to our results in Table 4. Thus, even in a minimum of 7-year follow-up, posterior-only approach has been proved safe and efficient in maintaining deformity correction. In addition, there was no significant difference in angle correction or maintaining correction between posterior-only approach and combined posterior-anterior approaches. Therefore, the posterior approach achieved equally good results compared to combined posterior-anterior approaches in terms of deformity correction and bone fusion. Moreover, satisfactory outcomes of indices including VAS, JOA score, ODI, and Kirkaldy-Willis functional outcomes at the final visit were demonstrated in all patients of both groups.

In consideration of a minimum of 7-year follow-up after lumbar and lumbosacral fusion surgery, ASD may occur with time. In this study, ASD occurred in 3 cases of group A (7.1%) and 2 cases of group B (5%) at the last visit, of which the incidence of ASD in both groups were much lower than that in degenerative spine disease (9.6% at 5-year follow-up) [35].

However, the posterior-only approach has limits. It was not applicable in cases with large multi-segment paravertebral abscesses or huge ilio-psoas abscesses, where an anterior debridement is necessary. In recent years, CT-guided percutaneous abscesses drainage and focal catheter infusion have been introduced as a new approach in the management of STB abscesses [36, 37]. In this study, a case with postoperative abscess formation in group A was successfully treated via this method, which may be a safe and effective way in adjuvant therapy combined with posterior approach [38, 39]. Moreover, for cases with severe destruction of three or more vertebral bodies due to difficult reconstruction of the spinal anterior column, combined anterior-posterior approaches were superior to posterior-only approach.

This study has two main limitations. First, its retrospective nature may have resulted in biased outcomes. Second, the sample size was relatively small. Therefore, prospective studies with larger samples are needed.

## Conclusions

Both combined posterior-anterior approaches and posterior-only approach can achieve satisfactory clinical and radiological outcomes of long-term in treating LSTB and LSSTB, while posterior-only approach can safely and effectively achieve lesion debridement, decompression, and stability reconstruction and maintenance with the advantages of minor surgical invasion, less bleeding, shorter surgery time and hospital stay, and fewer complications. So, posterior-only approach seemed to be superior to the combined posterior-anterior one.

## Data Availability

The datasets generated and/or analyzed during the current study are available from the corresponding author on reasonable request.

## Abbreviations

ASD: Adjacent segment degeneration
CRP: C-reactive protein
CT: Computed tomography
ESR: Erythrocyte sedimentation rate
JOA: Japanese Orthopaedic Association
LSSTB: Lumbosacral spinal tuberculosis
LSTB: Lumbar spinal tuberculosis
MRI: Magnetic resonance imaging
ODI: Oswestry Disability index
STB: Spinal tuberculosis
TB: Tuberculosis
UCLA: University of California at Los Angeles
VAS: Visual analogue scale

## References

[1] WHO: Global tuberculosis report 2018. World Health Organization (WHO) 2018.

[2] Pigrau-Serrallach Carlos, Rodríguez-Pardo Dolores (2012). Bone and joint tuberculosis. European Spine Journal.

[3] Liu Z, Wang J, Chen G-Z, Li W-W, Wu Y-Q, Xiao X, Zhang Y-L, Yang Y, Hu W-K, Sun Z-C (2019). Clinical characteristics of 1378 inpatients with spinal tuberculosis in general hospitals in South-Central China. Biomed Res Int.

[4] Liu Z, Zhang P, Zeng H, Xu Z, Wang X (2018). A comparative study of single-stage transpedicular debridement, fusion, and posterior long-segment versus short-segment fixation for the treatment of thoracolumbar spinal tuberculosis in adults: minimum five year follow-up outcomes. Int Orthop.

[5] Moon MS, Moon YW, Moon JL, Kim SS, Sun DH (2002). Conservative treatment of tuberculosis of the lumbar and lumbosacral spine. Clin Orthop Relat Res.

[6] Liu Z, Wang X, Xu Z, Zeng H, Zhang P, Peng W, Zhang Y (2016). Two approaches for treating upper thoracic spinal tuberculosis with neurological deficits in the elderly: a retrospective case-control study. Clin Neurol Neurosurg.

[7] Zhang P, Wei P, Wang X, Luo C, Xu Z, Hao Z, Zheng L, Zhang Y, Lei G. Minimum 5-year follow-up outcomes for single-stage transpedicular debridement, posterior instrumentation and fusion in the management of thoracic and thoracolumbar spinal tuberculosis in adults. Br J Neurosurg. 2016:1–6. 10.1080/02688697.2016.1206182 27387195

[8] Benli I, Kaya AE: , Anterior instrumentation in tuberculous spondylitis: is it effective and safe? Clin Orthop Relat Res 2007, 460(460):108-116. 10.1097/BLO.0b013e318065b70d 17452918

[9] Jain AK, Dhammi IK, Prashad B, Sinha S, Mishra P (2009). Simultaneous anterior decompression and posterior instrumentation of the tuberculous spine using an anterolateral extrapleural approach. J Bone Joint Surg Br.

[10] Wang X, Pang X, Wu P, Luo C, Shen X (2014). One-stage anterior debridement, bone grafting and posterior instrumentation vs. single posterior debridement, bone grafting, and instrumentation for the treatment of thoracic and lumbar spinal tuberculosis. Euro Spine J.

[11] Xu Z, Wang X, Shen X, Luo C, Zeng H, Zhang P, Peng W (2015). Posterior only versus combined posterior and anterior approaches for lower lumbar tuberculous spondylitis with neurological deficits in the aged. Spinal Cord.

[12] Bezer M, Kucukdurmaz FN, Kocaoglu B, Guven O (2005). Tuberculous spondylitis of the lumbosacral region: long-term follow-up of patients treated by chemotherapy, transpedicular drainage, posterior instrumentation, and fusion. J Spinal Disord Tech.

[13] Azimi P, Mohammadi HR, Montazeri A (2012). An outcome measure of functionality and pain in patients with lumbar disc herniation: a validation study of the Japanese Orthopedic Association (JOA) score. J Orthop Sci.

[14] van Hooff ML, Spruit M, Fairbank JC, Van LJ, Jacobs WC (2015). The Oswestry Disability Index (version 2.1a): validation of a Dutch language version. Spine.

[15] Ghiselli G, Wang JC, Hsu WK, Dawson EG (2003). L5-S1 segment survivorship and clinical outcome analysis after L4-L5 isolated fusion. Spine.

[16] Lee CK, Vessa P, Lee JK (1995). Chronic disabling low back pain syndrome caused by internal disc derangements. The results of disc excision and posterior lumbar interbody fusion. Spine.

[17] Nowakowski P, Delitto A, Erhard RE (1996). Lumbar spinal stenosis. Clin Ortho Related Res.

[18] Yao J, Jing L, Feng J, Jie M, Peng L, Jin L, Song W. The treatment progress of lumbosacral tuberculosis. Chinese J Clin. 2015.

[19] Myung-Sang M (2014). Tuberculosis of spine: current views in diagnosis and management. Asian Spine J.

[20] Zeng H, Wang X, Pang X, Luo C, Zhang P, Peng W, Wu P, Xu Z (2014). Posterior only versus combined posterior and anterior approaches in surgical management of lumbosacral tuberculosis with paraspinal abscess in adults. Eur J Trauma Emerg Surg.

[21] Li M, Du J, Meng H, Wang Z, Luo Z (2011). One-stage surgical management for thoracic tuberculosis by anterior debridement, decompression and autogenous rib grafts, and instrumentation. Spine J.

[22] Cavuşoğlu H, Kaya RA, Türkmenoğlu ON, Tuncer C, Colak I, Aydin Y (2008). A long-term follow-up study of anterior tibial allografting and instrumentation in the management of thoracolumbar tuberculous spondylitis. J Neurosurg Spine.

[23] Ma YZ, Cui X, Li HW, Chen X, Cai XJ, Bai YB (2012). Outcomes of anterior and posterior instrumentation under different surgical procedures for treating thoracic and lumbar spinal tuberculosis in adults. Int Orthop.

[24] Jie Z, Xiao Feng L, Tie Sheng H, Hui M, Zhi Ming C (2007). Anterior debridement and bone grafting of spinal tuberculosis with one-stage instrumentation anteriorly or posteriorly. Int Orthop.

[25] Bing W, Guohua L, We?Dong L, Ivan C: Anterior radical debridement and reconstruction using titanium mesh cage for the surgical treatment of thoracic and thoracolumbar spinal tuberculosis: minimium five-year follow-up. Turk Neurosurg 2011, 21(4):575-581. 22194119

[26] Wang B, Lv G, Liu W, Cheng I (2010). Anterior radical debridement and reconstruction using titanium mesh cage for the surgical treatment of thoracic and thoracolumbar spinal tuberculosis: minimium five-year follow-up. Turk Neurosurg.

[27] Zaveri GR, Mehta SS (2009). Surgical treatment of lumbar tuberculous spondylodiscitis by transforaminal lumbar interbody fusion (TLIF) and posterior instrumentation. J Spinal Disord Tech.

[28] Hong-Qi Z, Min-Zhong L, Lei G, Jin-Song L, Jian-Huang W, Jin-Yang L (2012). Surgical management by one-stage posterior transforaminal lumbar debridement, interbody fusion, and posterior instrumentation for lumbo-sacral tuberculosis in the aged. Arch Orthop Trauma Surg.

[29] Lehmer SM, Steffee AD, Gaines RW (1994). Treatment of L5-S1 spondyloptosis by staged L5 resection with reduction and fusion of L4 onto S1 (Gaines procedure). Spine.

[30] Wang LJ, Zhang HQ, Tang MX, Gao QL, Zhou ZH, Yin XH (2017). Comparison of three surgical approaches for thoracic spinal tuberculosis in adult: minimum 5-Year follow up. Spine.

[31] Talu Ufuk, Gogus Abdullah, Ozturk Cagatay, Hamzaoglu Azmi, Domanic Unsal (2006). The Role of Posterior Instrumentation and Fusion After Anterior Radical Debridement and Fusion in the Surgical Treatment of Spinal Tuberculosis: Experience of 127 Cases. Journal of Spinal Disorders & Techniques.

[32] Wang YX, Zhang HQ, Liao W, Tang M-X, Guo C-F, Deng A, Wu JH, Liu JY (2016). One-stage posterior focus debridement, interbody graft using titanium mesh cages, posterior instrumentation and fusion in the surgical treatment of lumbo-sacral spinal tuberculosis in the aged. Int Orthop.

[33] Zhang HQ, Lin MZ, Li JS, Tang MX, Guo CF, Wu JH, Liu JY (2013). One-stage posterior debridement, transforaminal lumbar interbody fusion and instrumentation in treatment of lumbar spinal tuberculosis: a retrospective case series. Arch Orthop Trauma Surg.

[34] Pang X, Shen X, Wu P, Luo C, Xu Z, Wang X (2013). Thoracolumbar spinal tuberculosis with psoas abscesses treated by one-stage posterior transforaminal lumbar debridement, interbody fusion, posterior instrumentation, and postural drainage. Arch Orthop Trauma Surg.

[35] Maruenda JI, Barrios C, Garibo F, Maruenda B (2016). Adjacent segment degeneration and revision surgery after circumferential lumbar fusion: outcomes throughout 15 years of follow-up. Eur Spine J.

[36] Hou X, Sun X, Zhang Z, Xie G, Zhang X: CT-guided percutaneous focal catheter infusion in treatment of spinal tuberculosis. Acta Orthopaedica Belgica 2015, 80(4). 26280722

[37] Li J, Huang X, Chen F, Dai F, Zhou Q, Luo F, Xu J, Zhang Z (2017). Computed tomography-guided catheterization drainage to cure spinal tuberculosis with individualized chemotherapy. Orthopedics.

[38] Hao Z, Zhang Y, Shen X, Luo C, Xu Z, Zheng L, Liu X, Wang X (2015). Staged treatment of thoracic and lumbar spinal tuberculosis with flow injection abscess. Int J Clin Experiment Med.

[39] Zou DX, Zhou JL, Zhou XX, Jiang XB (2017). Clinical efficacy of CT-guided percutaneous huge ilio-psoas abscesses drainage combined with posterior approach surgery for the management of dorsal and lumbar spinal tuberculosis in adults. Orthop Traumatol Surg Res.

